# Long term outcome and side effects in patients receiving low-dose I125 brachytherapy: a retrospective analysis

**DOI:** 10.1590/S1677-5538.IBJU.2015.0542

**Published:** 2016

**Authors:** Pieter Logghe, Rolf Verlinde, Frank Bouttens, Caroline Van den Broecke, Nathalie Deman, Koen Verboven, Dirk Maes, Luc Merckx

**Affiliations:** 1 Department of Urology, AZ St Lucas, Ghent, Oost-Vlaanderen, Belgium; 2 Department of Radiotherapy-Oncology, AZ St Lucas, Ghent, Oost-Vlaanderen, Belgium; 3 Department of Pathology, AZ St Lucas, Ghent, Oost-Vlaanderen, Belgium; 4 Department of Physics, AZ St Lucas, Ghent, Oost-Vlaanderen, Belgium

**Keywords:** Brachytherapy, Survival, Prostate-Specific Antigen, Prostatic Neoplasms

## Abstract

**Objectives:**

To retrospectively evaluate the disease free survival (DFS), disease specific survival (DSS),overall survival (OS) and side effects in patients who received low-dose rate (LDR) brachytherapy with I125 stranded seeds.

**Materials and methods:**

Between july 2003 and august 2012, 274 patients with organ confined prostate cancer were treated with permanent I125 brachytherapy. The median follow-up, age and pretreatment prostate specific antigen (iPSA) was 84 months (12-120), 67 years (50-83) and 7.8 ng/mL (1.14-38), respectively. Median Gleason score was 6 (3-9). 219 patients (80%) had stage cT1c, 42 patients (15.3%) had stage cT2a, 3 (1.1%) had stage cT2b and 3 (1.1%) had stage cT2c. The median D90 was 154.3 Gy (102.7-190.2).

**Results:**

DSS was 98.5%.OS was 93.5%. 13 patients (4.7%) developed systemic disease, 7 patients (2.55%) had local progression. In 139 low risk patients, the 5 year biochemical freedom from failure rate (BFFF) was 85% and 9 patients (6.4%) developed clinical progression. In the intermediate risk group, the 5 year BFFF rate was 70% and 5 patients (7.1%) developed clinical progression. Median nPSA in patients with biochemical relapse was 1.58 ng/mL (0.21 – 10.46), median nPSA in patients in remission was 0.51 ng/mL (0.01 – 8.5). Patients attaining a low PSA nadir had a significant higher BFFF (p<0.05). Median D90 in patients with biochemical relapse was 87.2 Gy (51 – 143,1). Patients receiving a high D90 had a significant higher BFFF (p<0.05).

**Conclusion:**

In a well selected patient population, LDR brachytherapy offers excellent outcomes. Reaching a low PSA nadir and attaining high D90 values are significant predictors for a higher DFS.

## INTRODUCTION

Prostate cancer is the most common malignancy in Belgian men, according to data from the Belgian Cancer Registry 2008. Increasing age, race and a positive family history are the most important risk factors (1). Due to PSA based screening, more low risk prostate cancers are detected. In patients with organ-confined disease, radical prostatectomy, low and high dose brachytherapy and external beam radiotherapy all have proven to have comparable outcomes in terms of biochemical relapse-free survival (2, 3). For this reason, patients often use treatment related morbidity to guide their personal preferences. With an aging population, more than half of the patients are now treated with radiotherapy. Compared to EBRT, brachytherapy is a simple outpatient procedure with quick recovery. With brachytherapy, high radiation dose can be locally delivered with a steep dose gradient in surrounding healthy tissues (4). Because the prostate gland is not removed in brachytherapy, follow-up based on biochemical control is more difficult than after radical prostatectomy. In this retrospective analysis, we wanted to evaluate the outcomes and side effects post-brachytherapy and try to identify predictors of these outcomes.

## MATERIALS AND METHODS

Between July 2003 and August 2012, 274 consecutive patients with localized prostate cancer were treated with permanent brachytherapy at the Ghent St. Lucas Hospital using I^125^ stranded seeds (Oncura I^125^ RAPID Strand™ implants, Arlington Heights, USA). Administered dose was 145Gy (Task Group 43 recommendation (5)). All patients were treated using the hybrid interactive image guided Mick technique (Mick Radionuclear Instruments Bronx, NY, USA) with the volume acquisition study, planning and seed implantation all taking place under a single general anesthesia. No AHT or EBRT was added previous to the treatment. Mean follow-up was 76 months (12-120). The median age was 67 years (50-83). Median iPSA was 7.8ng/mL (1.14-38) 219 (82%) had stage cT1c, 41 (15.4%) had stage cT2a, 3 (1.1%) had stage cT2b and 3 (1.1%) had stage cT2c. Median Gleason score and D90 was 6 (3-9) and 154.3Gy (102.7-190.2), respectively (Table-1).

**Table 1:** Characteristics of the patients who received LDR. Risk stratification was based on the D'Amico classification (PSA, Gleason and stage). The majority had a low risk prostate cancer (63.8%), one third had medium risk prostate cancer (32.1%).

Variable		n	%
**Clinical stage**			
	T1c	219	80
	T2a	42	15.3
	T2b, c	6	2.2
	No data	7	2.5
**Gleason score**			
	<7	195	71.2
	7	44	16
	>7	5	1.8
	No data	30	10.9
**PSA level**			
	<10	210	76.6
	10-20	58	21.16
	>20	6	2.18
**D'Amico classification**			
	Low risk	175	63.8
	Medium risk	88	32.1
	High risk	11	4

The risk stratification was based on the D'Amico classification (6). Low risk patients had stage T1-T2a disease, Gleason ≤6 or a PSA level ≤10ng/mL. Medium risk patients had Gleason 7, PSA level 10-20ng/mL or stage T2b. High risk patients had PSA level ≥20ng/mL, a Gleason score ≥8 or stage cT2c/T3a. No patients in our study had cT3a stage. 175 patients (63.8%) were classified as low risk, 88 (32.1%) were classified as medium risk and 11 patients (4%) were classified as high risk.

The median pretreatment IPSS was 4 (0-16). Median post residual volume (PVR), prostate volume and flow rate were 0mL (0-100), 28mL (15-52) and 14mL/s (6-38), respectively. Patients with an IPSS score of ≥10, signs of obstructive micturition (flow ≤10 mL/sec, PVR ≥150mL) and prostate volume ≥50mL were excluded from brachytherapy. No upfront AHT was administered to reduce the prostate volume. An alpha-blocker was given one week before the intervention and was continued 3 months postoperatively. Patients were given an enema and ciprofloxacin 500mg 2 hours before the procedure. A urinary catheter was placed intra-operatively and removed the next day.

Four weeks after implantation, post-implant CT dosimetry was performed. Calculated parameters were the percentage volume of the prostate receiving 90% and 100% of the prescribed dose and the amount of dose delivered to 90% of the prostate (V90, V100 and D90, respectively). Patients were evaluated every 3 months during the first year, every 6 months during the first 5 years and annually thereafter. Digital rectal examination (DRE) and PSA sampling were routinely performed each visit. Genito-urinary and rectal side-effects were assessed using the RTOG scoring scale.

Biochemical recurrence was defined as any PSA increase to >2ng/mL above the nadir value (ASTRO Phoenix definition) (1). PSA bounce was defined as a post-treatment rise with spontaneous return to pre-bounce levels. If there was biochemical recurrence, imaging (CT scan, bone scan) was used in selected cases to rule out systemic progression (bone metastases, lymph node involvement) and new prostate biopsies (PPB) were done. For the last 2 years, Choline PET-CT has been used to detect systemic progression in an early stage (7).

Logistic regression with the Wald Chi-Square test was used to evaluate the effect of different predictors on the disease free survival. Further analyses were conducted to see if we could identify risk factors responsible for side effects. A p-level <0.05 was considered statistical significant.

## RESULTS

Of the 274 patients treated with brachytherapy, 4 died of prostate cancer (DSS 98.5%). The OS was 93.5% (14 of 274 patients), the predominant mortality cause were other malignancies (lung, kidney, colon, liver). 13 patients (4.7%) developed systemic disease (bone metastases, lymph node involvement), 8 patients (2.9%) had local progression.

In 139 low risk patients, the 5-year biochemical freedom from failure rate (BFFF) was 85% and 10 patients (7.2%) developed clinical (systemic or local) progression. In the intermediate group, the 5 year BFFF rate was 70% and 5 patients (7.1%) developed clinical progression. In 9 high risk patients, the 5 year BFFF rate was 70%, 1 patient (11%) developed clinical progression.

A LHRH agonist or antagonist with or without anti-androgen therapy was administered when significant progression was detected (a fast raise in PSA and >2ng/mL above the nadir value or visualization of systemic progression on medical imaging). In our cohort, 33 patients (12%) received adjuvant AHT for local failure. If no systemic disease was present, a salvage radical prostatectomy was performed with or without lymphadenectomy depending on Gleason score, stage and PSA level. This procedure was performed in 7 patients (2.5%). No patients were given salvage EBRT, 1 patient had antalgic radiotherapy for a bone metastasis in the lumbar spine.

Of 216 patients with a minimum of 5 years follow-up, 177 (82%) reached their PSA nadir (nPSA) in the first 5 years. Median nPSA in patients with biochemical relapse was 1.58ng/mL (0.21-10.46), median nPSA in patients in remission was 0.51ng/mL (0.01-8.5). Logistic regression showed a significant (p<0.05) higher BFFF in patients reaching a low PSA nadir value and in patients with a lower pre-treatment PSA level. The number of implanted seeds was another predictor which was significantly higher in patients in remission (Table-2).

**Table 2:** Logistic regression with disease free survival after 5 years as outcome variable. The number of implanted seeds, PSA level pre-brachytherapy, PSA nadir and estimated D90 level on CT scan were significant predictors.

Variable	p-value predictor
Number of seeds	0.0265
PSA pre	0.0019
PSA nadir	<0.0001
Riskgroup LMH	0.0565
Gleason score	0.3737
D90inop	0.4693
D90CT	0.0189

Of 274 patients, 8 (2.7%) developed EUR requiring placement of a suprapubic catheter (CTCAE 4.0, grade 2). All patients had their suprapubic catheter removed in 4 weeks. Median IPSS and prostate volume in the retention group were 7.5 and 30mL, respectively. In the other patients, median IPSS was 5 and mean prostate volume was 29mL. The IPSS score was significantly higher (p<0.05) in patients who developed urinary retention, prostate volume was no significant predictor (p=0.3) (Table-3).

**Table 3:** Logistic regression with retention after LDR as outcome variable. The only factor that was a significant cause for retention was a high IPSS score.

Variable (predictor)	p-value predictor
Age	0.3105
IPSS	0.0007
Prostate volume	0.3105
Flow	0.6903
Residual	0.8455
D90inop	0.9157
D90CT	0.1202

Late (>3 months) urinary and rectal morbidity were evaluated based on the Radiation Therapy Oncology Group (RTOG) scoring system. 18 patients (6.5%) reported G2 frequency, 31 patients (11.2%) had G2 dysuria and 4 patients (1.4%) developed intermittent hematuria (G2) 2 patients (0.6%) had severe frequency (G3) 5 (1.8%) patients had G1 rectal toxicity and 3 (1%) patients had G2 rectal toxicity. None had G4 toxicity. No patient had persistent rectal symptoms or hematuria after 1 year. 4 patients (1.4%) lost a seed during micturition. 1 patient (0.3%) developed urethral stricture.

Mean IPSS and prostate volume in the patients with post treatment urinary morbidity was 5.01 and 29.4mL, respectively. In patients without urinary morbidity, mean IPSS was 3.9 and prostate volume was 28.6mL, which was not significantly lower.

The median D90 measured during the procedure was 154.3Gy (102.7-190.2). Patients in remission had a median D90 of 1564Gy (102.7-187.2) and median D90 in patients with biochemical relapse was 156.3Gy (110.5-190.2). However, median D90 values based on CT dosimetry 4 weeks later revealed a lower dose of 95.5Gy (51.1-162.4). Patients in remission had a significantly (p<0.05) higher D90 of 102.1Gy (51.4-162.4) than patients with biochemical relapse, mean D90 was 87.2Gy (51-143.1).

## DISCUSSION

**Figure 1:**
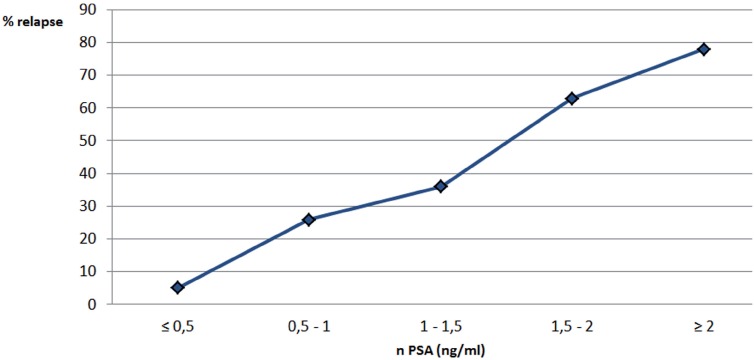
% relapse compared to nPSA level. Patients attaining a low PSA nadir have a significantly improved BFFF. When we compare the 5 year biochemical free survival in patients based on the PSA nadir, there is an almost linear correlation between the nadir and the relapse rate.

**Figure 2:**
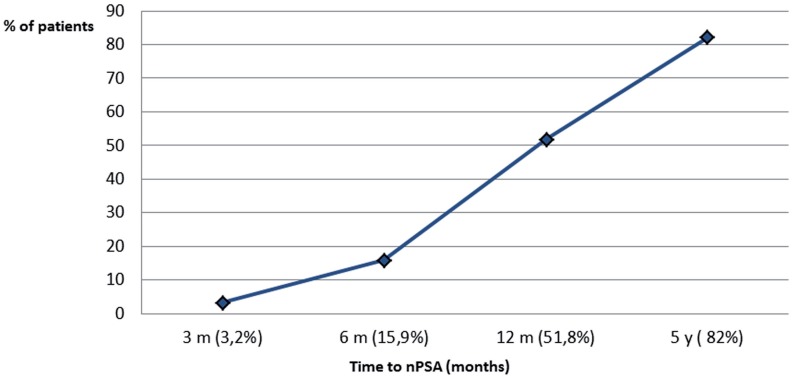
% patients reaching nPSA at specific intervals. 50% of the patients reached the nadir 1 year after brachytherapy, 82% of the patients reached the PSA nadir 5 years postoperatively.

In this retrospective analysis, we showed that patients attaining a low PSA nadir had a significantly improved BFFF. When we compared the 5-year biochemical free survival in patients based on the PSA nadir, there was an almost linear correlation between the nadir and the relapse rate (Figure-1). This confirms the findings of other studies (8). Eric et al. found that reaching a PSA nadir <0.5ng/mL was associated with a significant higher BFFF (9), which is similar to the findings of Leonardo et al., showing a better outcome when a PSA nadir <0.285ng/mL was reached at 1 year (10). Paoluzzi et al. stated that reaching a PSA nadir above 20% of the pretreatment value at 6 months was a worse prognostic factor (11). In our cohort, 82% of the patients had reached the PSA nadir at 5 years postoperatively (Figure-2).

**Figure 3:**
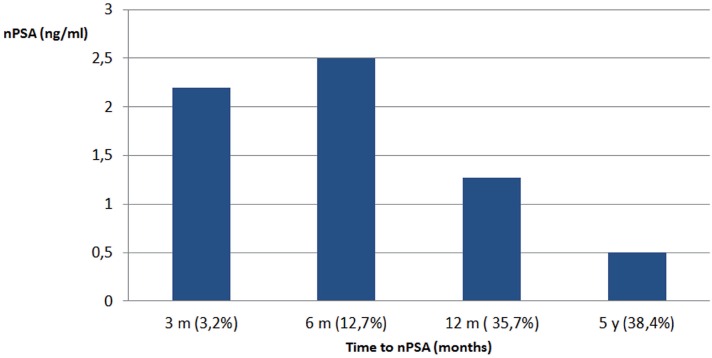
Mean nPSA values in different patients measured at specific time interval. Reaching the PSA nadir fast does not necessarily mean that the treatment is more efficient. Patients with a slowly declining PSA tended to reach a lower PSA nadir.

Reaching the PSA nadir fast does not necessarily mean that the treatment was more efficient with a better outcome. Patients with a slowly declining PSA tended to reach a lower PSA nadir (Figure-3). The BFFF in our population was comparable to other treatment modalities such as EBRT and radical prostatectomy (12) (Table-4). When we compared the post-brachytherapy BFFF to the other series in the literature, there were no important differences (12-17) (Table-5). In our cohort, BFFF rate in the high risk group was 70%, while the results in other studies showed higher relapse rates. As there were only 9 high risk patients, this result has to be interpreted cautiously.

**Table 4a:** 5 year biochemical freedom from failure rate published by Kupelian, D' Amico and Pound after radical prostatectomy in different risk groups.

Risk	Kupelian	D'Amico	Pound
Low	85%	83%	94-82%
Intermediate	65%	50%	72%
High	32%	28%	54%

**Table 4b:** 5 year biochemical freedom from failure rate published by Zelefski after external beam radiation therapy in different risk groups. The results are comparable with the results in table 4a.

Risk	Zelefski
Low	90%
Intermediate	70%
High	47%

**Table 5:** 5 year biochemical freedom from failure rate after brachytherapy. Results in the low risk group are similar in the different series and show a high BFFF. Blasko, Grado and our center achieved a high BFFF in the intermediate group. Relapse was high in the high risk group. As there were only 9 patients in the high risk group in our center, this data can underestimate the real relapse rate.

Risk	Blasko	Wallner	Grado	St. Lucas
Low	94%	100-80%	82%	85%
Intermediate	82%	45%	75%	70%
High	65%	39%	57%	70%‡

‡ Only 9 patients in this group.

Many studies stress the importance of a high biological effective dose (4, 12, 18) resulting in high D90 values. A D90 value of >140Gy showed an improved biochemical control (14). 91.7% of the patients received an intra-operative D90 >140Gy. We compared median intra-operative D90 values in our population but there was no significant (p=0.4) difference between patients with biochemical relapse (156.3Gy) and patients in remission (156.4Gy). Calculated D90 values based on CT dosimetry 4 weeks post-implantation showed a median D90 of 95.5Gy. Median D90 in the relapse group was 87.2Gy and D90 in the remission group was 102.1Gy, which is a significant difference (p<0.05). This confirms the findings in other centers of the positive correlation between BFFF and a high D90 on post treatment CT.

The reason for the important difference between the intra-operative D90 and post implant D90 is the difficulty to estimate the prostate volume on CT scan, with a considerable interobserver contour variability. Apex, base and periprostatic plexus are difficult to delineate on CT, measurements can exceed the ultrasound-volume by 20-40% (4). Furthermore, because of the brachytherapy the prostate is enlarged which causes an underestimation of the D90 if the scan is performed too early (<4 weeks) (19). For this reason, some authors advocate the use of MRI as post-implant dosimetry to achieve more accurate calculations (12, 20).

Four patients (1.4%) lost one or more seeds during micturition. Except for dosimetric purposes, no follow-up radiographs were taken so we have no data if other migration occurred. It has been shown that the most frequent site of seed migration is the chest and it occurs less frequently with stranded seeds than if loose seeds are used (21, 22). Migration is most common in the first 30 days postoperatively. All our patients were implanted with stranded I^125^ seeds.

**Figure 4:**
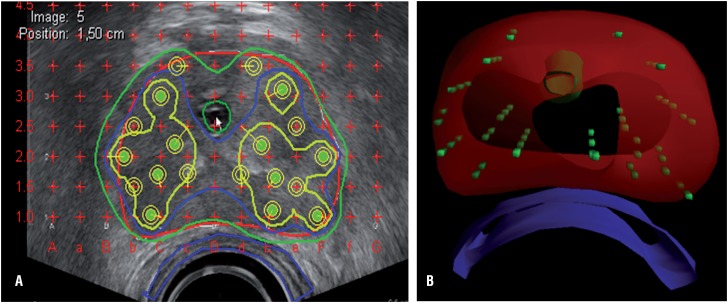
Left: Image of intra-operative planning on transrectal ultrasound. The colored lines mark the different areas where the radiation dose is similar. Urethra and rectum are marked to keep the radiation dose as low as possible in these regions. Right: 3D reconstruction of the seeds after implantation.

The implant process was based on the hybrid interactive Mick technique, no patients were preplanned (Figure-4). Preplanning means measurement of the prostate gland by ultrasound weeks before the procedure, giving the radiation oncologists ample time to perform the exact calculations and planning of the seeds. However, prostate size can differ because of swelling intra-operatively or shrinkage by AHT treatment (23). Advocates of the preplan method argue that due to the longer duration of intra-operative planning with loss of precious operation time, but this was refuted by Woolsey et al. (24). Thomas et al. showed superior results using intra-operative planning, with reduction of preplanning time, treatment time, number of needles used and an excellent dosimetric coverage (25). As more accurate radiation doses can be delivered using this method, Haim et al. found a slight elevation in urinary symptoms after intra-operative planning (26). Because of these higher doses, outcomes are better when intra-operative planning is used (27, 28).

When radiation therapy is used, the risk of secondary malignancies has always to be considered 10 patients (3.6%) developed a new primary tumor post-brachytherapy. Only 1 patient (0.3%) developed bladder cancer and underwent a cystoprostatectomy. The other patients had malignancies at a distance of the radiation field (lung, kidney, brain, transverse colon). Zelefsky et al. showed that after brachytherapy, the incidence of secondary malignancies was not significantly higher than in the control group. Risk factors appear to be related to tobacco smoking and patient age (29). Furthermore, tumors were not more aggressive than in the control group. When EBRT was used, the incidence of skin cancer did significantly increase (30).

In persons with organ confined disease, several treatment options are available. Because all have proven to have a similar outcome, (12, 14-17) patients tend to choose their treatment based on the expected side effects (2, 3). In brachytherapy, urinary, rectal and erectile problems are the most common toxicities. Initially the acute morbidity occurs, which is thought to be the result of a combination of local trauma and radiation damage. Dysuria, frequency, urgency, nocturia and a weak stream are common during the first months. About 90% of the patients will have a normalization of their urinary complaints 1 year post-brachytherapy (14). Late toxicity is less frequent (except for sexual dysfunction) and rarely persists past 10 years.

**Figure 5:**
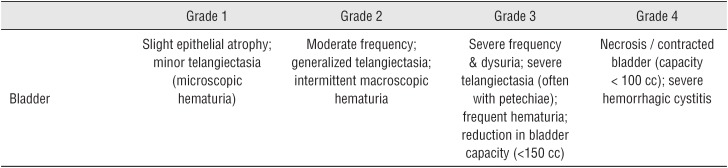
RTOG score, used to evaluate late urinary toxicity (>3 months). Only a minority of the patients had urinary complaints. Dysuria and frequency (grade 2) were the most common urinary side-effects of the treatment. Hematuria was only present in 1.4% of the patients.

**Figure 6:**
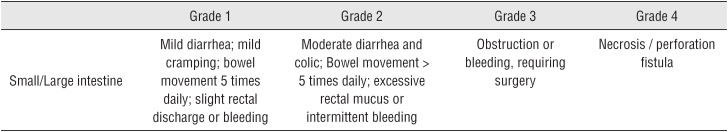
RTOG score, used to evaluate late rectal toxicity (> 3 months). In St Lucas, only 2.8% of the patients had rectal toxicity, none higher than grade 2.

Daphna et al showed a grade 1 and 2 rectal toxicity in 9.5% of the patients after brachytherapy with a peak at 8 months, all resolved in 3.5 years (31, 32). Pretreatment IPSS and prostatic volume were significant predictors of urinary toxicity. Brown et al. found the mean number of sources implanted and the total activity implanted to be correlated with the morbidity outcome (33, 34). In our cohort we evaluated both late urinary and rectal morbidity using the RTOG scale (Figures 5 and 6).

At 3 months, 19.7% of the patients had urinary toxicity, mostly grade 2. Only 0.6% had grade 3 symptoms, none had grade 4 urinary symptoms. 2.8% of the patients had grade 1 or 2 rectal toxicity, none had grade 3 or 4 rectal symptoms (Table-6). Dysuria and frequency were most common and treated by continuing the alpha-blocker treatment. Evaluation of other series shows a wide range of percentages, with all events varying from grade 1 to 3. None had grade 4 toxicity (Tables 7 and 8).

**Table 6:** RTOG late genito urinary and rectal toxicity (>3 months). No grade IV toxicity was seen, only a few patients had grade III toxicity. The results are comparable with other series.

Toxicity	Grade 1	Grade 2	Grade 3	Grade 4
**Rectal**	5 (1.8%)	3 (1%)	0	0
**Total genito-urinary**	0	53 (19.1%)	2 (0.6%)	0
**Frequency**	0	18 (6.5%)	2 (0.6%)	0
**Dysuria**	0	31 (11.2%)	0	0
**Hematuria**	0	4 (1.4%)	0	0

**Table 7:** Urinary toxicity after brachytherapy reported by Buckstein, Machtens, Daphna and Brown. No patients had grade IV toxicity. Low grade urinary toxicity is frequently seen but is self-limiting and 90% of the patients will have a normalization of their urinary complaints 1 year post-brachytherapy.

Author	Urinary G I	Urinary G II	Urinary G III	Urinary G IV
Machtens (14)	-	-	1-3	0
Buckstein (31)	-	4	3,6	0
Gelblum (33)	21.4	12.8	3	0
Brown (34)	37	37	6	0

**Table 8:** Rectal toxicity after brachytherapy published by Buckstein and Daphna. No high grade toxicity (grade IV) was reported. Most of the patients did not have any rectal toxicity. If toxicity was present, the peek was seen at 8 months, all resolved in 3.5 years.

Author	Rectal G I	Rectal G II	Rectal G III	Rectal G IV
Buckstein (31)	-	5.3	3	0
Gelblum (32)	9.4	6.6	0.5	0

In our cohort, prostate volumes >50mL were excluded from brachytherapy. The group with urinary comorbidity (53 patients; 19.1%) had a median prostate volume of 29mL, the prostate volume in the other group was 27mL. IPSS score was 3.5 in patients without urinary morbidity and 5 in the other group. Nicola et al. described a good postoperative flow rate and IPSS score in patients with a prostate volume up to 100mL (35). These findings were confirmed by Meyer et al., who stated that patients with a prostate volume >50mL had a similar postoperative IPSS score compared to volumes <50mL (36).

However, several studies mentioned prostate volume to be a significant factor of urinary retention after brachytherapy. Lee et al. found the number of needles and prostate volume as significant factors predicting for urinary retention after brachytherapy (37). Nicola et al. stated that the pre-operative IPSS score and the prostate volume were the strongest predictors for urinary retention (38). In our population, a significantly higher preoperative IPSS score was observed in the group with retention requiring placement of a suprapubic catheter (p<0.05). Prostate volume was no significant predictor (p=0.3). Median IPSS and prostate volume in the retention group were 7.5 and 30mL, respectively. In the other patients, median IPSS was 4 and mean prostate volume was 28mL. In all patients an alpha-blocker was initiated 1 week before the procedure and continued 3 months postoperative. Use of alpha-blockers was found to result in significantly less urinary morbidity and faster normalization of the IPSS. It had no impact on urinary retention (39).

Several studies found brachytherapy to have a negative impact on erectile function. Using the International Index of Erectile Function (IIEF), brachytherapy induced erectile dysfunction and occurred in 50% of the patients at 3 years; others mentioned a global decrease in all domains of the questionnaire 12 months post-brachytherapy (40, 41). Predictors for erectile dysfunction were mainly the radiation dose delivered to the proximal penis and the pre-implant IIEF score (14, 41, 42). Other factors were diabetes, age and hypertension. Merrick et al. stated that the use of a PDE-5 inhibitor improved potency outcomes post-brachytherapy (43). These results show the importance of minimizing the radiation dose to 50% of the penile bulb to less than 40% of the maximum dose, the dose to the crus should be less than 28% of the maximum dose (14, 41). Apart from these penile structures, Early et al. found that excessive radiation to the apical and peri-apical urethra was associated with a higher incidence of a urethral stricture (44). In our population only 1 patient (0.3%) developed an urethral stricture.

The major limitation of this study is that it is a retrospective analysis, without validated questionnaires. Follow-up was based on subjective reports written by physicians. Hence erectile dysfunction, IPSS scores and a longer standardized follow-up to evaluate the urinary and rectal toxicity postoperatively was not possible. Another shortcoming is the small number of subjects in certain subgroups.

## CONCLUSIONS

In a well selected patient population, low dose brachytherapy offers excellent outcomes. Survival rates are comparable with other treatment modalities. Attaining a low PSA nadir and a high D90 are important parameters to achieve minimal biochemical relapse rates. Urinary and rectal toxicity occurs but is often mild and selflimiting. Intra-operative planning with careful placement of the seeds is necessary to achieve a high D90 and at the same time avoiding the proximal penis, apical urethra and rectum.
